# Prevalence and Associated Factors of Maternal Depression and Anxiety Among African Immigrant Women in Alberta, Canada: Quantitative Cross-sectional Survey Study

**DOI:** 10.2196/43800

**Published:** 2023-02-20

**Authors:** Chinenye Nmanma Nwoke, Oluwagbohunmi A Awosoga, Sheila McDonald, Glenda T Bonifacio, Brenda M Y Leung

**Affiliations:** 1 Faculty of Health Sciences University of Lethbridge Lethbridge, AB Canada; 2 Department of Pediatrics and Community Health Sciences Faculty of Medicine University of Calgary Calgary, AB Canada; 3 Department of Women and Gender Studies University of Lethbridge Lethbridge, AB Canada

**Keywords:** African women, immigrant women, mental health, pregnancy, postpartum health, depression, anxiety

## Abstract

**Background:**

Although there is a significant body of evidence on maternal mental health, an inadequate focus has been placed on African immigrant women. This is a significant limitation given the rapidly changing demographics in Canada. The prevalence of maternal depression and anxiety among African immigrant women in Alberta and Canada, as well as the associated risk factors, are not well understood and remain largely unknown.

**Objective:**

The purpose of this study was to investigate the prevalence and associated factors of maternal depression and anxiety among African immigrant women living in Alberta, Canada up to 2 years postpartum.

**Methods:**

This cross-sectional study surveyed 120 African immigrant women within 2 years of delivery in Alberta, Canada from January 2020 to December 2020. The English version of the Edinburgh Postnatal Depression Scale-10 (EPDS-10), the Generalized Anxiety Disorder-7 (GAD-7) scale, and a structured questionnaire regarding associated factors were administered to all participants. A cutoff score of 13 on the EPDS-10 was indicative of depression, while a cutoff score of 10 on the GAD-7 scale was indicative of anxiety. Multivariable logistic regression was used to determine the factors significantly associated with maternal depression and anxiety.

**Results:**

Among the 120 African immigrant women, 27.5% (33/120) met the EPDS-10 cutoff score for depression and 12.1% (14/116) met the GAD-7 cutoff score for anxiety. The majority of respondents with maternal depression were younger (18/33, 56%), had a total household income of CAD $60,000 or more (US $45,000 or more; 21/32, 66%), rented their homes (24/33, 73%), had an advanced degree (19/33, 58%), were married (26/31, 84%), were recent immigrants (19/30, 63%), had friends in the city (21/31, 68%), had a weak sense of belonging in the local community (26/31, 84%), were satisfied with their settlement process (17/28, 61%), and had access to a regular medical doctor (20/29, 69%). In addition, the majority of respondents with maternal anxiety were nonrecent immigrants (9/14, 64%), had friends in the city (8/13, 62%), had a weak sense of belonging in the local community (12/13, 92%), and had access to a regular medical doctor (7/12, 58%). The multivariable logistic regression model identified demographic and social factors significantly associated with maternal depression (maternal age, working status, presence of friends in the city, and access to a regular medical doctor) and maternal anxiety (access to a regular medical doctor and sense of belonging in the local community).

**Conclusions:**

Social support and community belonging initiatives may improve the maternal mental health outcomes of African immigrant women. Given the complexities immigrant women face, more research is needed on a comprehensive approach for public health and preventive strategies regarding maternal mental health after migration, including increasing access to family doctors.

## Introduction

### Background

Canada is an important destination country for international migrants and is known for having one of the most diverse populations in the world, mainly driven by immigration. Specifically, immigrant women in Canada represent a significant and fast-growing group. According to the 2011 National Household Survey data, there are about 3.5 million immigrant women in Canada, representing 21% of the female population [1], and 66% of immigrant women were from racialized (ie, African) backgrounds, while 19.3% of the total female population in Canada were from racialized backgrounds [1]. The number of women immigrating to Canada has grown since 1980, with a larger proportion of racialized women from developing countries.

Additionally, between 2011 and 2016, the number of people who identified as racialized increased significantly across Canada [2]. This mosaic of individuals in Canada from different cultures presents a unique and evolving challenge for health care provision, especially for racialized immigrant women. Because the current Canadian immigration policy is dictated primarily by economic preferences and skilled immigration priorities, the number of highly educated professional immigrant women is growing, even though they continue to immigrate to Canada primarily as dependents, either in the family class category or as the spouse of a principal applicant [3,4]. The effects of this influx of racialized immigrant women into Canada is likely reflected in the provision of their health care, including after childbirth [4].

African immigrant women represent a sizeable and rapidly growing group, yet they are underrepresented in health research on maternal mental health. Between 2006 and 2011, over 145,700 people emigrated from Africa to Canada, and African immigrants currently comprise 13.6% of the Canadian immigrant population, which is relatively more than the proportion of European immigrants (11.9%) [5,6]. In spite of the growing presence of African immigrants in Canada, maternal mental health research focusing on African immigrants is sparse [7]. Very little effort has been made to examine African women’s immigration and the risk of developing depression and anxiety following childbirth. To date, there are no prevalence statistics for maternal depression or anxiety among African immigrant women in Alberta and Canada. In addition, with the increasing number of African immigrants, no research in Canada has examined the prevalence and determinants of maternal depression and anxiety for African immigrant populations. Maternal depression and anxiety in the postpartum period have been shown to have negative consequences [8,9]. For immigrants, however, these consequences are greater because they face other vulnerabilities associated with the stressful life event of migration to a new country [8,9].

The postpartum maternal mental health of immigrants as a whole appears to be particularly poor; specifically, the occurrence and associated risks of depression and anxiety appear to be twice as high in recent immigrant women when compared with their Canadian counterparts [10]. Moreover, it is generally accepted that depression and anxiety occur more frequently in individuals who are emotionally and socially disadvantaged [11]. Canadian studies have also concluded that immigrant women from Africa (determined by region of origin) exhibited the highest rates of emergency cesarean section, higher risk of preterm births, and higher rates of infant mortality, which are all risk factors for maternal depression and anxiety disorders [12,13].

African immigrants are on the rise in Canada, are at higher risk of mental health issues in the postpartum period, and are at higher risk of birth complications, yet they remain an understudied group for maternal depression and anxiety [7]. With an increasing influx of African immigrants into Alberta and Canada, the occurrence of maternal depression and anxiety in this population warrants consideration at the population health level. Additionally, as societies become more diverse like Canada, researchers and clinicians are continually challenged to enhance their understanding of perinatal and postpartum mental health care [7]. Highlighting the importance of more research will create a solid foundation for better treatment and coping strategies for the target population.

### Objective

The principal aim of this study was to examine the prevalence of maternal depression and anxiety within 2 years after delivery among African immigrant women. The need to better understand and serve the African community in Canada is becoming more evident; it is now more critical than ever for service providers and researchers to become aware of the magnitude and risk factors of depression and anxiety as integral components of the overall health of African immigrant women.

Recognizing that maternal depression and anxiety are understudied among African immigrant women in Canada, the purpose of this study was 2-fold, and it aimed to answer the following research questions: (1) What is the prevalence of maternal depression and anxiety among African immigrant women in Alberta up to 2 years postpartum? and (2) What is the association of demographic (age, income, working status, home ownership, education, marital status, years since immigration, and age at the time of immigration) and social (family/friends in the city, sense of belonging, community group membership, availability of mental health services, satisfaction with the settlement process, and access to a regular medical doctor) risk factors with the symptoms of maternal depression and anxiety among African immigrant women in Alberta up to 2 years postpartum?

## Methods

### Participants

This was a cross-sectional study carried out from January to December 2020 in Alberta, Canada. We recruited 120 African immigrant mothers aged ≥18 years with infants aged ≤2 years. Participants were identified and recruited in partnership with community organizations, immigrant-serving organizations, and religious leaders, as well as through the snowballing recruitment strategy. Participants were included if they met the following criteria: (1) were aged ≥18 years, (2) were Alberta residents, (3) previously had a live birth and currently have an infant aged ≤2 years, and (4) self-identified as African. Participants who answered “yes” to all 4 questions regarding the inclusion criteria were deemed eligible to participate in the full study. Following this inclusion questionnaire, there was a prompt to access the 63-item study questionnaire, which had questions regarding the Edinburgh Postnatal Depression Scale-10 (EPDS-10); General Anxiety Disorder-7 (GAD-7) scale; and sociodemographic, socioeconomic, social network, health utilization, and acculturation variables. The questionnaire was pilot tested and administered online through the Qualtrics platform or through a request for a paper-based questionnaire package. In both instances, informed consent was obtained prior to accessing the questionnaire. Detailed recruitment strategies for this study are given elsewhere [14].

### Survey Administration

The study questionnaire was programmed using the Qualtrics survey platform. Before data collection, informed consent was obtained from the prospective study participants through the Qualtrics survey platform. All the participants were informed that they had the right to withdraw from the study at any time without any consequences; this was explicitly noted in the letter of consent. Participants who met the study inclusion criteria and provided consent were automatically able to access the study questionnaire via a link to Qualtrics. The introduction to the questions included the study rationale in layman’s terms, a description of the survey structure and indicators of the survey progress (using static statements), the anticipated time commitment, and the researcher’s contact information. The survey was made accessible to eligible participants until the end of the study recruitment period. The Qualtrics platform allows partially completed survey data to be saved; thus, participants were able to save and continue the survey if any interruptions arose.

The option of having a paper-based version of the study questionnaire mailed to the participants’ residence was offered to participants with no access to the internet. These packages included the recruitment memo outlining the purpose of the study, a paper-based version of the study consent form, a paper-based version of the study questionnaire, and a list of resources and support services from Alberta Health Services available to mothers in Alberta. Participants were provided with a postage-paid return envelope and were asked to return the questionnaire package at their earliest convenience. If the questionnaire package was not returned after 3 weeks, reminders were sent via the method through which the participant had expressed interest.

By using the primary method of survey administration for this study (ie, on the web via Qualtrics), in addition to providing alternative administration options at community and cultural events and having a paper-based version mailed out, the study was able to reach a suitable sample size of 120 African immigrant mothers.

### Outcome Variables

We assessed and analyzed the prevalence of maternal depression and anxiety among study respondents. Maternal depression was measured using the EPDS-10. The EPDS-10 takes 5 minutes to administer and is readable at a third-grade level [15]. Responses are scored on a 4-point scale from 0 to 3 according to the severity of the symptoms, and total scores range from 0 to 30, with higher scores indicative of a higher risk for postpartum depression [15]. In this study, a cutoff score of 13 was indicative of depression [16-18]. The EPDS-10 has a sensitivity (the ability of the tool to correctly identify respondents with depression symptoms) of 86% and a specificity (the ability of the tool to correctly identify respondents without depression symptoms) of 78% [15].

Maternal anxiety was measured using the GAD-7 scale, a self-administered questionnaire used as a screening tool and severity measure for anxiety disorder [19]. The GAD-7 score is calculated by assigning scores of 0, 1, 2, or 3 to the response categories and adding together the scores for the 7 questions; scores of 5, 10, and 15 are taken as the cutoff points for mild, moderate, and severe anxiety, respectively [20]. Using the threshold score of 10, the GAD-7 scale has a sensitivity of 89% and a specificity of 82% [20].

### Other Covariates

Other covariates of interest in the analysis were grouped into demographic factors and social factors. Demographic factors included maternal age (≤30 years or >30 years), age of the most recent infant (0-12 months or ≥12 months), total household income (CAD $59,999 or less [US $44,999 or less] or CAD $60,000 or more [US $45,000 or more]), working status (on maternity leave or not on maternity leave), home ownership (own or rent), highest level of maternal education (bachelor’s degree or less, or advanced degree), marital status (married/common-law or not married/never married), years since immigration (0-5 years or ≥5 years), and age at the time of immigration (0-17 years [child] or ≥18 years [adult]). Social factors included family/friends in the city (yes or no), sense of belonging (strong or weak), community group membership (yes or no), availability of mental health services (fair/poor or excellent/good), satisfaction with the settlement process (satisfied or dissatisfied), and access to a regular medical doctor (yes or no).

### Data Analysis

Preliminary analyses consisted of descriptive frequencies and percentages, and tests of association (chi-square test and Fisher exact test) were used to determine differences between selected categorical variables. Variables associated with the outcomes at a level of *P*<.10 in unadjusted analyses were entered into the final multivariable models after assessing for the presence of multicollinearity. Multivariable logistic regression was performed to identify predictive variables significantly associated with maternal depression and anxiety, and the odds ratios (ORs) and 95% CIs have been presented. The confounding effects of covariate variables on the study outcome variables were assessed at each stage of the model building process; any variable that caused a change of 20% or more in the regression coefficient of the primary exposure of interest was considered a confounder and was retained in the model. Statistical significance was set at a *P* value of <.05. To achieve 80% power at an alpha of .05, a minimum sample size of 102 was needed. All analyses were performed in SAS Enterprise version 7.1 (SAS Institute).

### Ethics Approval

The study was approved by the University of Lethbridge Human Participant Research Committee on December 11, 2019 (HPRC protocol number 2019-116). Participants were provided with a study information sheet and asked to sign a consent form prior to accessing the study questionnaire. Participants were also entered into a raffle draw to win 1 of 5 CAD $20 (US $15) gift cards as an incentive for participation.

## Results

Among 120 African immigrant mothers who were enrolled in the study, 27.5% (33/120) screened positive for maternal depression with the EPDS-10 at a cutoff of 13 and 12.1% (14/116) screened positive for maternal anxiety with the GAD-7 scale at a cutoff of 10.

Table 1 compares the demographic characteristics of African immigrant women with and without symptoms of maternal depression and anxiety. The proportion of respondents who were younger (≤30 years) was higher among those with maternal depression than among those without maternal depression (18/32, 56% vs 29/82, 35%; *P*=.04). The proportion of respondents who were on maternity leave was higher among those with maternal depression than among those without maternal depression (16/32, 50% vs 22/85, 26%; *P*=.01). The proportion of respondents who were married was lower among those with maternal depression than among those without maternal depression (26/31, 84% vs 80/84, 95%; *P*=.05). No statistically significant differences between these respondent groups were observed for age of the most recent infant, total household income, home ownership, maternal education, years since immigration, and age at the time of immigration. The proportion of respondents who were nonrecent immigrants (≥6 years since immigration) was higher among those with maternal anxiety than among those without maternal anxiety (9/14, 64% vs 24/92, 26%; *P*=.01). No statistically significant differences between these respondent groups were observed for maternal age, age of the most recent infant, total household income, working status, home ownership, maternal education, marital status, and age at the time of immigration (Table 1).

Table 2 presents the association between social factors and probable maternal depression and anxiety. The presence of friends in the city, sense of belonging in the local community, satisfaction with the settlement process, and access to a regular medical doctor were significantly associated with maternal depression in African immigrant mothers. The proportion of respondents with absence of friends in the city was higher among those with maternal depression than among those without maternal depression (10/31, 32% vs 7/84, 8%; *P*=.001). Moreover, the proportion of respondents with a weak sense of community belonging was higher among those with maternal depression than among those without maternal depression (26/31, 84% vs 40/84, 48%; *P*<.001). The proportion of respondents who were satisfied with the settlement process was lower among those with maternal depression than among those without maternal depression (17/28, 61% vs 64/81, 79%; *P*=.06). The proportion of respondents with a lack of access to a regular medical doctor was higher among those with maternal depression than among those without maternal depression (9/29, 31% vs 6/81, 7%; *P*=.002). Furthermore, the proportions of respondents with absence of family in the city, lack of group participation, and fair/poor ratings on the availability of mental health services in the community were higher among those with maternal depression than among those without maternal depression (21/31, 68% vs 43/84, 51%; 23/31, 74% vs 53/83, 64%; and 8/28, 29% vs 18/74, 24%, respectively); however, these findings were not significant (Table 2).

The presence of friends in the city, sense of belonging in the local community, and access to a regular medical doctor were significantly associated with maternal anxiety in African immigrant mothers. The proportion of respondents with absence of friends in the city was higher among those with maternal anxiety than among those without maternal anxiety (5/13, 38% vs 12/100, 12%; *P*=.01). Moreover, the proportion of respondents with a weak sense of community belonging was higher among those with maternal anxiety than among those without maternal anxiety (12/13, 92% vs 53/100, 53.0%; *P*=.01). The proportion of respondents with no access to a regular medical doctor was higher among those with maternal anxiety than among those without maternal anxiety (5/12, 42% vs 10/96, 10%; *P*=.003). Furthermore, the proportions of respondents with absence of family in the city, lack of group participation, dissatisfaction with their settlement process in Canada, and fair/poor ratings on the availability of mental health services in the community were higher among those with maternal anxiety than among those without maternal anxiety (9/13, 69% vs 54/100, 54%; 11/13, 85% vs 64/99, 65%; 5/12, 42% vs 23/95, 24%; and 4/12, 33% vs 22/88, 25%); however, these findings were not significant (Table 2).

Tables 3 and 4 present the adjusted ORs (aORs) and 95% CIs from the multivariable logistic analyses for respondents with maternal depression and anxiety compared to those without maternal depression and anxiety, according to demographic and social characteristics. Regarding the covariates associated with maternal depression, the presence of friends in the city was a protective factor, controlling for the other factors. Respondents with friends in the city were less likely to develop maternal depression symptoms when compared with those with no friends in the city (aOR 0.19, 95% CI 0.05-0.71; *P*=.01). Younger respondents had higher odds of reporting maternal depression symptoms when compared with older respondents (aOR 5.57, 95% CI 1.74-17.82; *P*=.004). Moreover, respondents on maternity leave had higher odds of reporting maternal depression symptoms when compared with those not on maternity leave (aOR 4.56, 95% CI 1.49-13.99; *P*=.001). Moreover, respondents with access to a regular medical doctor were less likely to report maternal depression symptoms when compared with those with no access to a regular medical doctor (aOR 0.10, 95% CI 0.02-0.45; *P*=.003) (Table 3).

Sense of belonging and regular access to a medical doctor were significant protective factors for maternal anxiety. Respondents with a strong sense of belonging in the local community were less likely to report maternal anxiety symptoms when compared with those with a weak sense of community belonging (aOR 0.12, 95% CI 0.02-0.99; *P*=.04). Moreover, respondents with access to a regular medical doctor were less likely to report maternal anxiety symptoms when compared with those with no access to a regular medical doctor (aOR 0.19, 95% CI 0.05-0.74; *P*=.02) (Table 4).

**Table 1:** Demographic characteristics of African immigrant women with and without symptoms of maternal depression and anxiety.

Factors^a^		Maternal depression				Maternal anxiety		
		Participants with maternal depression (EPDS-10^b^ ≥13), n/N (%)	Participants without maternal depression (EPDS-10 <13), n/N (%)	*P* value	Participants with maternal anxiety (GAD-7^c^ ≥10), n/N (%)		Participants without maternal anxiety (GAD-7 <10), n/N (%)	*P* value
**Maternal age**				.04				.56
	≤30 years	18/32 (56)	29/82 (35)		7/14 (50)		56/96 (58)	
	≥31 years	14/32 (44)	53/82 (65)		7/14 (50)		40/96 (42)	
**Age of the most recent infant**				.74				.90
	0-12 months	17/32 (53)	48/85 (56)		8/14 (57)		56/101 (55)	
	≥12 months	15/32 (47)	37/85 (44)		6/14 (43)		45/101 (45)	
**Total household income (CAD$)^d^**				.20				.51
	≤59,999	11/32 (34)	40/84 (48)		5/14 (36)		45/100 (45)	
	≥60,000	21/32 (66)	44/84 (52)		9/14 (64)		55/100 (55)	
**Working status**				.01				.82
	Maternity leave	16/32 (50)	22/85 (26)		5/14 (36)		33/101 (33)	
	Not on maternity leave	16/32 (50)	63/85 (74)		9/14 (64)		68/101 (67)	
**Home**				.34				.77
	Rent	24/33 (73)	54/85 (64)		10/14 (71)		66/102 (65)	
	Own	9/33 (27)	31/85 (36)		4/14 (29)		36/102 (35)	
**Maternal education**				.90				.27
	Bachelor’s degree or less	14/33 (42)	35/85 (41)		4/14 (29)		44/100 (44)	
	Advanced degree	19/33 (58)	50/85 (59)		10/14 (71)		56/100 (56)	
**Marital status**				.06				.28
	Not married	5/31 (16)	4/84 (5)		2/13 (15)		7/100 (7)	
	Married/common law	26/31 (84)	80/84 (95)		11/13 (85)		93/100 (93)	
**Years since immigration**				.42				.01
	0-5 years	19/30 (63)	57/80 (71)		5/14 (36)		68/92 (74)	
	≥6 years	11/30 (37)	23/80 (29)		9/14 (64)		24/92 (26)	
**Age at immigration**				.94				.46
	<18 years (child)	5/28 (18)	15/81 (19)		1/12 (8)		19/95 (20)	
	≥18 years (adult)	23/28 (82)	66/81 (81)		11/12 (92)		76/95 (80)	

^a^Variables with *P*<.10 were included in the multivariable model.

^b^EPDS-10: Edinburgh Postnatal Depression Scale-10.

^c^GAD-7: General Anxiety Disorder-7.

^d^A currency exchange rate of CAD $1=US $0.75 is applicable.

**Table 2:** Social characteristics of African immigrant women with and without maternal depression and anxiety.

Factors^a^		Maternal depression				Maternal anxiety		
		Participants with maternal depression (EPDS-10^b^ ≥13), n/N (%)	Participants without maternal depression (EPDS-10 <13), n/N (%)	*P* value	Participants with maternal anxiety (GAD-7^c^ ≥10), n/N (%)		Participants without maternal anxiety (GAD-7 <10), n/N (%)	*P* value
**Presence of family in the city**				.11				.30
	No family in the city	21/31 (68)	43/84 (51)		9/13 (69)		54/100 (54)	
	Family in the city	10/31 (32)	41/84 (49)		4/13 (31)		46/100 (46)	
**Presence of friends in the city**				.001				.01
	No friends in the city	10/31 (32)	7/84 (8)		5/13 (38)		12/100 (12)	
	Friends in the city	21/31 (68)	77/84 (92)		8/13 (62)		88/100 (88)	
**Sense of belonging**				<.001				.01
	Weak	26/31 (84)	40/84 (48)		12/13 (92)		53/100 (53)	
	Strong	5/31 (16)	44/84 (52)		1/13 (8)		47/100 (47)	
**Group participation**				.30				.21
	Not a member	23/31 (74)	53/83 (64)		11/13 (85)		64/99 (65)	
	Member	8/31 (26)	30/83 (36)		2/13 (15)		35/99 (35)	
**Availability of mental health services**				.66				.50
	Fair/poor	8/28 (29)	18/74 (24)		4/12 (33)		22/88 (25)	
	Excellent/good	20/28 (71)	56/74 (76)		8/12 (67)		66/88 (75)	
**Satisfaction with settlement**				.06				.19
	Dissatisfied	11/28 (39)	17/81 (21)		5/12 (42)		23/95 (24)	
	Satisfied	17/28 (61)	64/81 (79)		7/12 (58)		72/95 (76)	
**Access to a regular medical doctor**				.002				.003
	Yes	20/29 (69)	75/81 (93)		7/12 (58)		86/96 (90)	
	No	9/29 (31)	6/81 (7)		5/12 (42)		10/96 (10)	

^a^Variables with *P*<.10 were included in the multivariable model.

^b^EPDS-10: Edinburgh Postnatal Depression Scale-10.

^c^GAD-7: General Anxiety Disorder-7.

**Table 3:** Multivariable logistic regression analysis of demographic and social factors associated with maternal depression in African immigrant women.

Covariate		aOR^a^	95% CI	*P* value
**Maternal age**				
	≤30 years	5.57	1.74-17.82	.004
	≥31 years (reference)	N/A^b^	N/A	N/A
**Working status**				
	Maternity leave	4.56	1.49-13.99	.001
	Not on maternity leave (reference)	N/A	N/A	N/A
**Presence of friends in the city**				
	Friends in the city	0.19	0.05-0.71	.01
	No friends in the city (reference)	N/A	N/A	N/A
**Access to a regular medical doctor**				
	Yes	0.10	0.02-0.45	.003
	No (reference)	N/A	N/A	N/A

^a^aOR: adjusted odds ratio.

^b^N/A: not applicable.

**Table 4:** Multivariable logistic regression analysis of demographic and social factors associated with maternal anxiety in African immigrant women.

Covariate		aOR^a^	95% CI	*P* value
**Access to a regular medical doctor**				
	Yes	0.19	0.05-0.74	.02
	No (reference)	N/A^b^	N/A	N/A
**Sense of belonging**				
	Strong	0.12	0.02-0.99	.04
	Weak (reference)	N/A	N/A	N/A

^a^aOR: adjusted odds ratio.

^b^N/A: not applicable.

## Discussion

### Principal Findings

The results of this study demonstrated that maternal depression and anxiety are important features in African immigrant women within 2 years after giving birth. Almost one-third (33/120, 27.5%) of participants screened met the cutoff score for maternal depression, which is higher than what was previously reported in the literature for immigrant women and other immigrant groups in Australia [21], Canada [22-25], and Norway [26]. Moreover, 12.1% (14/116) met the criteria for increased anxiety symptoms, which is similar to the findings in previous studies [27-29]. It is evident that African immigrant women are exposed to many factors following childbirth, which can impact their maternal mental health, such as lack of social support, weak sense of belonging in the local community, and economic factors.

The prevalence of maternal depression has varied in studies conducted in different countries, with different methodologies, sample sizes, and inclusion and exclusion criteria. Specifically, the prevalence found in this study is high compared to that in most other studies in the field of maternal depression among immigrant women. A similarly high rate of 25.5% was found in a study on postnatal depression conducted among immigrant populations (Vietnamese and Indonesian) in Taiwan [30]; in this study, however, the findings should be viewed with caution because of the difficulties in validating the conceptual and semantic equivalence of translated instruments. Our study did not use translated versions of the EPDS-10 and GAD-7 scale. Although there is a scarcity of studies on the prevalence of maternal depression among African immigrant women, other studies have found marked lower rates of depression among immigrant mothers. In Canada, 4 different studies found rates of 12.6% [22], 13.2% [23], 20.0% [25], and 20.5% [24]. All 4 studies excluded those not able to answer in majority languages (English and French).

The high rate of maternal depression found in this study could also be linked to the fact that the majority of respondents with depression were recent immigrants (19/30, 63%). This contradicts both the immigrant paradox phenomenon, where immigrants who have just moved to a host country have better health outcomes than the majority population, and the acculturation paradox, where longer residency in a country is linked to poorer health outcomes than the majority population [31]. In this study, most of the variables followed the pattern of the immigrant paradox. However, with regard to maternal depression, a marked difference in prevalence was noted between recent immigrants with 0-5 years of residency (19/30, 63%) and long-term immigrants with ≥6 years of residency (11/30, 37%). These findings suggest that the prevalence of maternal depression was higher among recent immigrants than among nonrecent immigrants, thus contradicting the theory of the immigrant paradox.

Three different reasons for the increased rates of maternal depression seen in many studies on immigrant women have been suggested, and they include (1) sociocultural differences, (2) difficult economic situation for new immigrants, and (3) consequences of migration, especially premigration stress [32,33]. All of these aforementioned reasons could be used as valid arguments for expecting a higher rate of maternal depression in this sample, which did occur.

Furthermore, there is sufficient evidence to suggest that age is one of the most important factors affecting maternal mental health [34-36], yet the relationship between maternal depression or anxiety and age in African immigrant women has not been adequately studied. In this study, the effect of age on maternal mental health outcomes was identified, with lower rates of depression among women who gave birth at an advanced maternal age (≥35 years) than among women who gave birth at a younger age (additional analysis). This corroborates the findings of studies showing that a higher and more stable socioeconomic status often characterizes women of advanced maternal age [35-37] and could confer psychological benefits among women. This could be a possible reason why African immigrant women aged ≥35 years in the study sample had lower rates of both maternal depression and anxiety. In other studies, being a woman of advanced maternal age was also a protective factor for depression, even after controlling for mode of conception and known risk factors for maternal depression [38,39].

For maternal anxiety, however, the rate of 12% in this study is comparable to the rates in other studies on Mexican immigrant women in the United States (18%) [29], Chinese immigrant women in Canada (18.4%) [28], and African immigrants in the United States (12.4%) [27]. Maternal anxiety as a whole is an understudied area among African immigrant women, and thus, there is a scarcity of comparable literature. Nonetheless, in this study, the rate of maternal anxiety was lower than the rate of maternal depression. This is in part due to the perception that anxiety might be more culturally acceptable than depression [40] or anxiety might be less culturally acceptable and thus underreported among African immigrant women in Alberta.

Strong sense of belonging in the local community and access to a regular medical doctor were protective factors against maternal anxiety among African mothers. Practitioners should therefore assess African immigrant women living in Alberta for their sense of belonging/integration in the local community to identify women at risk of developing anxiety during pregnancy and after childbirth. Ultimately, the growing body of research on maternal mental health shows that depression and anxiety are often comorbid conditions. In this study, a chi-square test was performed to determine the correlation between the categorical variables of maternal depression and anxiety. There was sufficient evidence to show that the symptoms of maternal depression impact the likelihood of maternal anxiety and vice versa (*P*<.001). However, the extent or magnitude of this impact in African immigrant women warrants further studies.

Notably, social factors emerged as important determinants of maternal mental health outcomes in African mothers in Alberta. Respondents with friends in the city were less likely to report symptoms of maternal depression than those without friends in the city. According to Levitt et al [41], “the capacity of individuals to cope with transitional circumstances is facilitated by the presence of social support.” Thus, having friends living in the same city may be indicative of higher levels of social support and social capital, which may decrease the risk of maternal depression after childbirth among African mothers. Furthermore, the sense of belonging was a significant predictor of reporting symptoms of maternal anxiety. This corroborates findings from other studies conducted on immigrant populations, where having a strong sense of belonging in the local community was a protective factor for self-reporting symptoms of depression and anxiety [9,42]. This is also consistent with theorizing that social identity protects against mental illness through several social and psychological mechanisms. For immigrant women, a sense of belonging in the local community reflects whether they feel accepted, secure, and included in their adopted country [43,44]. For African immigrant mothers specifically, newcomer initiatives that foster a strong sense of community are a promising strategy to consider, as the social ties that accompany the sense of belonging are protective factors for maternal anxiety. Future studies could further explore the sense of belonging in the home country as a foundation to the sense of belonging in Canada after migration.

The study results support the presence of a strong association between having a regular medical doctor and symptoms of maternal depression and anxiety; respondents with access to a regular medical doctor were less likely to have symptoms of both maternal depression and anxiety in comparison to those with no access to a regular medical doctor. Family doctors are viewed as a gateway to maternal mental health services and play a key role in the care and management of mental health [45,46]. In addition, this study confirmed the findings of other studies from Canada showing that among those who seek mental health care, the majority consult with their family doctor [9,47]. The Canadian Strategy for Mental Health proposes that primary health care services be expanded to integrate specialized mental health care services in order to address the gaps in the mental health system [45]. Thus, family doctors who predominantly serve African women or immigrant women as a whole seem appropriate for this role expansion.

### Limitations

The limitations of this study were, for the most part, related to the cross-sectional quantitative research study design. This study relied upon cross-sectional survey data, and as a result, no inferences could be made about the cause-and-effect relationship between variables. All statistics reported are associations, and caution is needed in their application and interpretation, especially concerning temporality, as there may be residual confounding due to unknown or unmeasured confounders such as immigration type/class, ethnicity, and precarious employment. Moreover, the transferability and application of the findings to other settings/cultural groups are important issues owing to the sample size and representativeness of the sample.

Although the sample consisted of a variety of African immigrant women from different backgrounds, African mothers who responded to the study may differ from those who did not respond. In particular, given the topic of the survey, women who had depressive or anxiety symptoms may be more likely to respond than those who did not have these symptoms. Nonetheless, this study can be used as a baseline exploratory study for future applied research on African mothers and their maternal mental health. Further studies are needed to determine the applicability of the results to the larger African immigrant population in Alberta and Canada as a whole.

Additionally, as in all cross-sectional studies, this study only provides a snapshot of maternal depression and anxiety among African mothers in Alberta in a specific time and space. The variations in the prevalences of maternal depression and anxiety over time and their impacts on mental health are better examined with a longitudinal research design. Maternal depression and anxiety symptoms, and other variables of interest in this study were based on self-reported data, which can be subject to recall bias and social desirability bias. Nonetheless, the use of self-reported data as a strategy in studies on sensitive topics (ie, mental health) is valid and sometimes more appropriate than the use of administrative data.

### Implications

This study has implications for both clinical practice and future areas of research on African immigrant women with young infants. The findings from this study contribute to our knowledge of the disparities in maternal mental health outcomes among African mothers within 2 years after giving birth. The study results have shown that African mothers’ maternal mental health and well-being are associated with a variety of factors, including maternal age, social support (having friends in the same city of residence), sense of belonging in the local community, and having access to a regular medical doctor.

Immigration, Refugees, and Citizenship Canada’s settlement programs play a major role in accelerating social and economic integrations, which in turn influence African immigrant women’s overall health and maternal mental well-being. For example, this study found that having friends in the same city of residence and having a strong sense of belonging in the local community were associated with a decreased likelihood of experiencing maternal mental health outcomes. One of the central priorities of Canada’s settlement program is to encourage participation of immigrants in all aspects of Canadian social life, and it is designed to meet immigrants’ immediate needs by providing orientation and referral services and facilitating access to social, health, and recreational facilities. Therefore, these programs can play a significant role in supporting African immigrant women’s settlement and integration into Canadian society with the continued support of resettlement services directed to meet the needs of African immigrant women, especially their maternal mental health needs after migration.

### Conclusion

This study is possibly the first study in Alberta to examine maternal depression and anxiety outcomes among African mothers with infants aged 2 years or under. The findings of this study were generally consistent with the findings of studies conducted in other parts of the world [27-30]; however, there were some substantial variations. The prevalence of maternal depression was high, and it was found that 27.5% (33/120) of African immigrant women in the sample had symptoms of depression. The prevalence of maternal anxiety was 12.1% (14/116), and a similar prevalence has been reported in other studies [27-30]. The findings also highlight the importance of African mothers having strong social connections in the city and regular access to a medical doctor. More adequately powered research with a large sample size of African immigrants is warranted to further assess maternal depression and anxiety, and their risks.

Ultimately, the results of this study underscore the need for an increased understanding of this group of individuals in the population as they navigate the maternal mental health care system in Alberta and Canada. Further mixed method or qualitative studies need to be conducted to gain a more in-depth and nuanced understanding of the domains not examined in this study, for example, stigma, cultural adjustment issues, and underemployment of African immigrants, and how these may have cumulative impacts on maternal mental health.

## Abbreviations

aOR: adjusted odds ratio
EPDS-10: Edinburgh Postnatal Depression Scale-10
GAD-7: Generalized Anxiety Disorder-7
OR: odds ratio

## Data Availability

The data sets generated and analyzed during this study are not publicly available because of limitations of the ethics approval application but can be obtained from the corresponding author on reasonable request and with permission from the University of Lethbridge Human Participant Research Committee.
